# Supplementary material for: A biallelic variant in GORASP1 causes a novel Golgipathy with glycosylation and mitotic defects
Source: Life Sci Alliance. 2025 Feb 11;8(4):e202403065. doi: 10.26508/lsa.202403065 (PMC11814487; doi:10.26508/lsa.202403065)
Supplement: Supplementary file 1 [file LSA-2024-03065_Supplemental_Data_1.docx]

**Supplementary Material**

1. **Full description of the patient’s phenotype**

The phenotype associates sensorineural deafness, learning disabilities, white matter abnormalities, muscles contractures, severe myopia with degenerative vitreoretinopathy, truncal obesity and morphological features.

*Consanguineous family*

This 19 years and 6 months old patient is the third child of first cousin Tunisian parents without family history.

*No antenatal and neonatal events*

The pregnancy was unremarkable, with normal prenatal ultrasounds and fetal movements. Delivery took place uneventfully by scheduled cesarean section at 39 weeks of gestation due to narrow pelvis and scarred uterus. Apgar score was normal (10/10 at respectively 1 and 5 min). Birth weight was 3.6 kg, length was 48 cm, and head circumference was 35.5 cm. During the neonatal period, no feeding or swallowing problems were noted.

*Normal Psychomotor development during the first years of life*

Motor development during the first year of life was normal with head control acquired at two months, a stable sitting position at five and a half months, and independent walking at 14 months. The patient had good social interactions and achieved day and night continence at two years. The first stage of language development was normal with disyllables acquired at 10 months, first words at 2 years, but first sentences were not acquired at 3 years.

*Progressive sensorineural deafness*

Concerns began in the first preschool year (3-4 years) when the teacher reported that the child did not speak at school and did not like noise. Chronic serous otitis with conducting hearing loss was diagnosed. The patient underwent tonsillectomy and adenoidectomy with placement of a transtympanic ventilation tube placement at 4 years of age. However, brainstem auditory evoked potentials (BAEP) showed a wave V at 40 dB with normal latency and negative acoustic emissions. Follow-up at 5 years confirmed, by audiometry, moderate bilateral sensorineural hearing loss (-62 dB) in the right ear and more severe (-77 dB) in the left ear at 4000 Hz. The articulatory difficulties were linked to velopharyngeal insufficiency (Dr Martine Francois, Othorinolaryngology Department, Robert Debré Hospital, Paris, France). Between 6 and 11 years of age, deafness increased with a loss of -80 dB on both sides at 4000 Hz and -50 dB at 1000 Hz. Sound amplification has improved language and has so far enabled understanding and expression of language in the daily life, but special education for deaf children was necessary after the age of 7. Nasal speech has remained throughout evolution without drooling or swallowing difficulties.

Due to the hearing impairment, the patient underwent a brain MRI which showed a normal appearance of the inner ear structures but revealed an abnormal signal from the sus tentorial white matter. The patient was then referred and followed in the child neurology department (Reference Center for Leukodystrophies and Leukoencephalopathies; LEUKOFRANCE).

*Learning disabilities*

Learning disabilities not linked to deafness became clear in elementary school with difficulties in reading, writing and counting. He was included in a specific institution for children with deafness and learning disabilities at the age of 9. Impulsivity and hyperactivity with attention deficit have been successfully treated with methylphenidate up to 14 years of age. He recently obtained a professional aptitude certificate in plumbing. Cognitive assessments carried out at 6 years (WPSI-II) , 9 years (WISC –IV), and 13years and 8 months (WISC-V) confirmed poor verbal performances (CVI 68) with difficulties in fluid reasoning (FRI 79) and working memory (WMI 76) capacities but a normal visuospatial index (VSI 102) and processing speed (PSI 83). At 17 years and 8 months, specific written language tests for deaf children confirmed the persistence of a severe dyslexia and dysorthographia.

The head circumference curve remains in the normal range (50 p). Broad base gait and mild instability with eyes closed were the only neurological signs noticed throughout the evolution of the disease with absence of pyramidal and cerebellar dysfunctions

*White matter abnormalities*

The initial brain MRI, performed at the age of 5, revealed diffuse, almost symmetrical and heterogeneous hyperintensities in T2-weighted and fluid-attenuated inversion recovery (FLAIR) of the frontoparietal white matter, with predominance in the periventricular area, extending to the centrum semiovale, involving to a lesser degree the subcortical white matter, unevenly, but with preservation of the U-fibers. The lesions presented a mild T1 hypointensity and facilitated diffusion on ADC (apparent diffusion coefficient) maps. Interestingly, the white matter tracts in the corpus callosum, in bilateral internal capsules (anterior/posterior limbs), as well as the projections in the optic radiations were preserved. The basal ganglia showed normal morphological and signal, except for a few bilateral, periventricular microcysts (2-3 mm in size) located in the caudate nuclei (head and body). Findings in the posterior fossa were unremarkable, notably no cerebellar or vermis atrophy was found. MRI was supplemented by a single-voxel spectroscopy (1H-MRS/ short TE:30 ms and long TE:136 ms) in the periventricular and parietal left white matter lesions which revealed at long TE, a minimal reversed peak of lactate, a normal peak (according to the age) of N-acetylaspartate and myo-inositol, and a slight increase in the peak of choline. This MRI pattern was strongly suggestive of a leukodystrophy but not specific for a known pathology. Three MRI follow-up studies were performed over the following years (6.5 years, 9.4 years, 14.5 years), to assess the progression of abnormal findings. Subsequent MRI studies demonstrated a significant reduction in T2/FLAIR signal indicating a decrease in the extent of white matter lesions. The tiny periventricular cysts were stable, no new lesion appeared and the initial mild and non-specific alterations in spectroscopy were normalized, consistent with each age. In addition, the patient underwent complementary spine MRI follow-up with no observed medullary abnormality.

Somatosensory evoked potentials performed at 10 years were normal in the upper limbs after median nerve stimulation. After stimulation of the tibial nerve, central conductions (N22-P39) were in the upper normal range (mean 16 ms) more to the right (19.3 ms ) than to the left (18.1 ms) (Dr Hala Nasser, Neurophysiology, Robert Debré Hospital, Paris, France).

Blood counts, hemostasis, electrophoresis of hemoglobin, immunoglobulins, iron, ferritin, transferrin, lipid analysis, liver and kidney functions throughout the evolution remained perfectly normal. An thorough metabolic workup performed at 6 years was normal including urine organic acids, blood and urine amino acids chromatography, sialic aciduria, sulfatiduria, redox ratio in the fasting state and 1 hour after meal, acyl-carnitines and N-glycome in serum, carnitine in blood and urine, very long chain fatty acid, uric acid, glycosaminoglycans and pteridines in urine, glycosylation of the transferrin by electrophoresis, arylsulfatase A activity in the leukocytes. NGS analysis of a panel of 169 genes involved in leukodystrophies and leukoencephalopathies was negative. CSF analysis including lactates, pyruvates, proteins, glucose and cytology was also normal.

*Muscles contractures*

The first complaints concerned muscle pain in the legs particularly at night with stiffness in the morning upon rising (up to 1 hour) associated with frequent falls and walking fatigue during the day. Gabapentin and L-carnitine clearly improved the symptoms. Falls became rare after age 10, mainly when L-carnitine was disrupted. Morning stiffness decreased at the same time.

Episodes of exercise intolerance occurred when he started playing handball after the age of 15, mainly as cramps in the thighs and calves a few hours after the exercise with difficulty in climbing the stairs for a few hours. Acute painful muscle contractures of the thighs associated with bilateral knotted vastus lateralis for 10 minutes were reported on two occasions. The M

muscles appeared hypertrophic and stiff upon contraction, particularly in the calves and biceps on the right side, whereas the patient is left dominant. Muscle testing was always normal (score 5) with the exception of a slight decrease in the vastus lateralis (score 4). Triceps amplitude was bilaterally limited without worsening over time. Cinematic walking parameters improved over time from 10 to 19 years. Serum creatine kinase, aldolases, lactic dehydrogenase and myoglobinuria were always normal but never measured during an acute phase. EMG and nerve conduction velocities performed at 6 and 14 years were normal.

*Progressive high myopia with vitreoretinopathy*

Ophthalmological evaluations at 7 and 10 years were normal with normal electroretinography (ERG) and evoked potentials (VEP). Myopia developed rapidly in 6 months at the age of 12, reaching – 6.5 in both eyes. During a routine examination at 12 years 6 months, the ocular fundus revealed signs of lattice degeneration of the retina with holes associated with vitreous condensation suggestive of a Stickler syndrome (Dr Florence Metge, Adolphe Rotschild ophthalmological Foundation, Paris, France). Laser therapy was performed under general anesthesia twice over the next 2 months with good results. ERG and VEP, 2 years later, were normal. No complaints existed. At the last examination (18 years and 7 months), the bilateral vitreous opacities were increased but the retina and myopia (-7 diopters) remained stable. The lens was perfectly normal during evolution. NGS analysis of a panel of genes involved in Stickler syndrome was negative.

*Obesity*

At age 5, height (112 cm) and weight (22 kg) were in the upper normal range. The weight curve increased rapidly between 5 and 10 years of age. At 10 years and 6 months, with a weight of 55 kg, the body mass index (BMI) reached the threshold of 30 for obesity (International Obesity Task force). Obesity was essentially truncal with a pseudo gynecomastia. BMI decreased after 15 years to reach the upper limit of normal (25) at 18 years (weight 75 kg; height 173cm). However truncal obesity remained. Puberty was slightly delayed (15-17 years). Thyroid (T3,T4, TSH) and sexual hormones (FSH-LH, testosterone and antimullerian hormone) were normal.

*Morphological abnormalities*

Mild facial features were observed between the ages of 6 and 19 years: an elongated face with micrognathia, a prominent nose, downward slanting palpebral fissures with mild ptosis, thick eyebrows with synophrys, low seat ears, low frontal hairline and open mouth. Cervico dorsal kyphosis, ulna valgus, genu valgum with recurvatum of the knees were observed with a mild joint laxity.

During the adolescence (age 13), we noticed on the MRI of the spine progressive irregularities of the vertebral endplates at the level of 3 consecutive thoracic vertebrae (T7-T9), associated with anterior wedge deformation of a vertebral body (T8), without signal abnormality. The image was thought to be consistent with Scheuermann’s disease, also known as juvenile kyphosis or vertebral epiphysitis. Skeletal X-Rays further showed a coxa valga and a bone thinning of the medial part of the distal epiphysis of the radius, bilaterally. Dry skin was present with chronic eczema-like lesions predominant in the creases. At age 11, a skin cyst was rapidly growing in the left temporal region of the face. The anatomo-pathological analysis after surgical removal concluded that it was a pilomatrixoma. Echocardiography, abdominal and vesicorenal echography were normal.

1. **Detailed Genetic information**

Whole-genome trio sequencing revealed four rare variants, homozygous in the patient, in *TRAF4* (NM_004295.3; c.794 C>T, p.(Ala265Val)), *GAS2L2* (NM_139285.3; c.395T>C, p.(Met132Thr)), *GABRR1* (NM_002042:c.1406T>C p.(Leu469Ser)) and *GORASP1*. Variants in *TRAF4* (Tnf Receptor-Associated Factor 4) and *GAS2L2* (Growth Arrest-Specific 2-Like 2) were excluded as they were also present in the homozygous state in the healthy brother, and therefore did not segregate with the disease. The *GABRR1* variant co-segregated with disease in the family and was absent from the gnomAD variant database (gnomAD v4.1) but was predicted benign by AlphaMissense, the most powerful AI-powered model that evaluates missense variants based on protein structural predictions. Furthermore, the expression profile of *GABRR1* did not correlate with the patient's multiple tissue phenotype, and the gene was associated with susceptibility to schizoaffective bipolar disorder (10.1002/ajmg.b.31108), i.e. with no relation to our patient's phenotype. The remaining candidate variant was c.1170_1171del p.(Asp390Glufs*18) in the ubiquitously expressed *GORASP1* gene (NM_031899.4). The identified variant was absent in the homozygous state in gnomAD (gnomAD v4.1), predicted to cause a frameshift and a premature termination codon in the Golgi protein GRASP65. Since several other Golgi proteins have been associated with neurodevelopmental disorders whose symptoms matched fairly well to our patient's phenotype, the *GORASP1* variant was considered a relevant candidate.
